# Sleepless in Germany: psychometric validation of a German version of the Bedtime Procrastination Scale

**DOI:** 10.3389/fpsyg.2026.1918123

**Published:** 2026-09-08

**Authors:** Christina Ewert, Antonia Winkler, Samuel Tomczyk

**Affiliations:** 1 Department of Psychology, University of Potsdam, Potsdam, Germany; 2 Institute and Policlinic of Medical Psychology and Medical Sociology, University of Rostock, Rostock, Germany; 3 German Center for Child and Adolescent Health (DZKJ), Greifswald/Rostock, Rostock, Germany

**Keywords:** bedtime procrastination, German adaptation, procrastinating, psychometric validation, scale validation, self-control, sleep, sleep quality

## Abstract

**Introduction:**

Bedtime procrastination refers to delaying bedtime despite the absence of external constraints and awareness of potential negative consequences. It is linked to impaired sleep, reduced well-being, and self-regulatory difficulties. Although the Bedtime Procrastination Scale (BPS) is most widely used, no validated German version is available. This study aimed to translate, culturally adapt, and evaluate the psychometric properties of the German Bedtime Procrastination Scale (BPS-G).

**Methods:**

A sample of 548 German-speaking adults completed the BPS-G and measures of procrastination, self-control, affect, personality, life satisfaction, and sleep-related outcomes. Exploratory and confirmatory factor analyses were conducted to examine the factor structure. Reliability was assessed using McDonald’s omega and a six-week test–retest design. Convergent, discriminant, criterion, incremental, and predictive validity were evaluated using correlational and regression analyses.

**Results:**

Item analyses indicated satisfactory item characteristics and excellent discriminatory power. Exploratory factor analysis supported a one-factor solution explaining 59% of the variance. Confirmatory factor analysis largely supported the unidimensional structure (CFI = 0.94, TLI = 0.92, SRMR = 0.04). Internal consistency was excellent (*ω* = 0.94), and test–retest reliability was high (*r* = 0.80). Bedtime procrastination was positively associated with general procrastination and negative affect and negatively associated with self-control, conscientiousness, and positive affect. Higher bedtime procrastination predicted lower life satisfaction, poorer sleep quality, and shorter sleep duration. Gender differences in bedtime procrastination were not statistically significant.

**Discussion:**

The findings provide evidence for the reliability and validity of the BPS-G. The BPS-G offers a useful tool for assessing bedtime procrastination in German-speaking populations.

## 1 Introduction

In an age defined by perpetual connectivity and overstimulation, many individuals resist the very rest they actually crave: Each night, they scroll, stream, or simply linger—delaying bedtime long past their intended hour, despite knowing the consequences. This voluntary but irrational delay, known as *bedtime procrastination* (Kroese et al., 2014), has far-reaching implications for sleep health, cognitive functioning, and psychological well-being, similar to other forms of sleep deprivation. Studies report a prevalence of high bedtime procrastination of about 20% in university students aged 18 to 27 (Shukla and Andrade, 2023), with higher prevalence in older adolescents and emerging adults, making it a relevant public health concern. It is considered a domain-specific manifestation of general procrastination (e.g., Kroese et al., 2014) and captures the tendency to postpone going to bed without external constraints, leading to sleep loss, circadian misalignment, and impaired next-day functioning. Thus, it shifts the focus from *whether* one procrastinates to *when*, underlining the importance of situatedness of health and risk behaviors (such as procrastination).

Procrastination in general is among the most prevalent forms of dysfunctional self-regulation, characterized as the voluntary delay of an intended action despite anticipating negative outcomes (Steel, 2007). It affects an estimated 46% of university students in academic contexts (Solomon and Rothblum, 1984) and approximately 10% of adults in the general population (Ferrari et al., 1995). Chronic procrastination has been associated with poorer academic and occupational performance, elevated stress, and detrimental health and financial outcomes (Sirois, 2007; Steel, 2007). However, bedtime procrastination represents a domain-specific manifestation of this pattern—concerned not with *whether* one acts, but *when* (Kroese et al., 2014). It captures the tendency to postpone going to bed without external constraints, leading to sleep loss, circadian misalignment, and impaired next-day functioning.

Empirical studies across cultures consistently demonstrate the adverse consequences of bedtime procrastination. It predicts shorter sleep duration, delayed sleep onset, lower sleep efficiency, greater fatigue, and poorer next-day performance, even when controlling for chronotype, workload, and environmental factors (Hill et al., 2022; Kadzikowska-Wrzosek, 2021; Zhao et al., 2024) as well as is generally associated with increased negative affect as well as higher levels of neuroticism (Tang, 2024; Zhao et al., 2024) Longitudinal and daily-diary studies confirm that higher bedtime procrastination is associated with worse subjective sleep quality and daytime tiredness (Kühnel et al., 2018; Schmidt et al., 2024), while stress and negative affect increase the likelihood of postponing bedtime (Nauts et al., 2019). Moreover, psychological resources such as self-compassion and resilience have been shown to buffer—but not eliminate—the negative effects of bedtime procrastination on sleep and well-being (Sirois et al., 2019; Rasouli et al., 2025). These findings position bedtime procrastination as a key target for sleep health promotion and behavioral interventions. To address procrastination, intervention research shows that cognitive-behavioral therapy strengthening self-control and emotion regulation is effective, as it supports self-management and the uptake and maintenance of healthy alternatives to procrastination (Van Eerde and Klingsieck, 2018).

However, most of this evidence stems from non-German contexts. To advance culturally sensitive sleep research and behavioral interventions, a validated German measure is urgently needed.

In a recent diary and wearable-based study among German university students, for example, daily stress predicted bedtime procrastination, which in turn mediated poorer subjective sleep quality and next-day alertness (Kühnel et al., 2018). Personality factors such as low conscientiousness, high neuroticism, and low self-control further contribute to bedtime procrastination (Carlson and Williams, 2025; Nauts et al., 2019). Despite increasing attention to these processes, no psychometrically validated instrument currently exists in German, limiting the comparability of findings and the development of culturally grounded interventions. A German translation has been performed by Bernecker and Job (2020), who found evidence for validity in negative correlations between bedtime procrastination and self-control, sleep duration, and sleep quality. However, no examination of the factor structure of the translated BPS has been conducted to this day. Thus, this work aims to extend existing findings regarding the German version of the Bedtime Procrastination Scale by exploring if the BPS-G has a similar factor structure to the original BPS while also further inspecting the psychometric properties of the scale.

The Bedtime Procrastination Scale (BPS), developed by Kroese et al. (2014), remains the most widely used measure of this construct. The original English version demonstrated excellent internal consistency (Cronbach’s *α* = 0.92) and strong construct validity, correlating negatively with self-regulation and sleep duration and positively with fatigue and general procrastination. Since then, the BPS has been translated and validated in several languages, each confirming its factorial stability and reliability across cultures: The Spanish version (BPS-Sp) showed high internal consistency (*α* = 0.90) and excellent test–retest reliability (*r* = 0.87) among nursing students, indicating temporal stability and cross-linguistic equivalence (Brando-Garrido et al., 2022); the Chinese BPS demonstrated strong reliability (α = 0.89) and a robust one-factor structure confirmed via CFA, predicting both poor sleep quality and psychological distress (Ma et al., 2022; Cai et al., 2022); the Japanese version (BPS-J) exhibited similar psychometric soundness (α = 0.88), with evidence for convergent validity (e.g., self-control) and measurement invariance across gender (Hazumi et al., 2024); the Turkish adaptation further supported its structural validity, reporting internal consistency of α = 0.93 (Demir Kösem et al., 2024). Together, these findings confirm that bedtime procrastination is a cross-culturally stable construct, measurable through a brief, reliable self-report instrument.

Cultural factors—such as social norms around rest, evening routines, and technology use—may influence how bedtime procrastination manifests and should thus be reflected in assessment tools. The present study therefore aims to translate, culturally adapt, and psychometrically validate the German version of the Bedtime Procrastination Scale (BPS-G). Specifically, we will examine its factor structure, internal consistency, convergent and criterion validity, and test its associations with theoretically related constructs, such as procrastination or conscientiousness. As stated in our preregistration, we assume that (1) the BPS-G represents a unidimensional construct and therefore has a one-factor structure, (2) there is a moderate positive relation between bedtime procrastination and (a) general procrastination, (b) neuroticism, and (c) negative affect, (3) there is a moderate negative relation between bedtime procrastination and (d) self-control, (e) conscientiousness, and (f) positive affect. (4) there is no substantial association between bedtime procrastination and the Big Five factors (g) openness, (h) agreeableness and (i) extraversion. To complete the validation of the BPS-G, criterion validity will be investigated by inspecting the relations between bedtime procrastination and health outcomes like sleep quality and sleep duration as well as satisfaction with life. Research has shown that bedtime procrastination is linked to poor sleep quality and shorter sleep duration (Herzog-Krzywoszanska and Krzywoszanski, 2019), making it an important predictor of sleep outcomes. Considering this, we assume that (5) bedtime procrastination can predict satisfaction with life, sleep quality and number of hours slept. Correlations should exceed *r* = 0.30 and *r* = −0.30 to be substantial, according to Cohen (1988). To further add insights to the psychometric properties of the BPS-G, incremental validity, predictive validity and retest reliability will also be assessed exploratively, in addition to our preregistration.

Thus, establishing a robust and culturally sensitive German version will enable rigorous research on the mechanisms, correlates, and health outcomes of bedtime procrastination and provide a foundation for future prevention and intervention efforts targeting sleep-related self-regulation.

## 2 Materials and methods

### 2.1 Procedure

The study was preregistered in November 2025 before data collection, see https://aspredicted.org/74x2pf.pdf. Because of the observational nature of the study, none of the relevant scales were subject to any manipulation. Relevant data were collected via an online survey posted on the platform *SoSci survey* (Leiner, 2025). After they agreed to a declaration of consent, study subjects could participate in the study from November 8th, 2025, to November 27th, 2025, for the first measurement point (T1). Completing the questionnaire took about 30 min. Data for the second measurement point (T2) were collected 6 weeks later from January 7th, 2026, to January 20th, 2026, in a 30-min procedure again posted on *SoSci survey*.

### 2.2 Participants

The plan was to collect at least 500 participants at T1 to achieve a sufficient number of cases for factor analysis, in line with recommendations (Boateng et al., 2018). In total, 617 participated in the study, with 587 valid cases. 110 participated in the survey at T2. To participate in the study, subjects had to be at least 18 years of age and able to read and write in German. Participants who did not provide responses for the BPS were excluded, reducing the number of participants to 553. Furthermore, another five participants were excluded because they showed too fast completion times (2.0 standard deviations below the mean).

If they completed the second measurement point, participants had the opportunity to engage in a draw of ten gift cards from Green Lifestyle worth ten euros (approx. $11.50 US) each. Furthermore, they received an evaluation of their personality in terms of an above average, below average or average expression of the Big Five dimensions.

The remaining sample for T1 (*N* = 548) was 69% female, with a mean age of 34.41 years (*SD* = 15.16). 43.4% were students and 34.9% employees. *N* = 96 participants (71% female, with a mean age of 35.21 years, *SD* = 14.24) completed the survey at T2.

### 2.3 Measures

The following section gives an overview of the scales applied in the survey that were used for analyses regarding the validation of the German version of the BPS (for more detailed information, see Supplementary materials).

#### 2.3.1 Bed time procrastination German

The German version of the Bedtime Procrastination Scale was developed in a seminar group of the Master’s program in Psychology at the University of Potsdam adapted from the original BPS by Kroese et al. (2014) (German items see Table 1). The translation was performed by several native German speakers. Then, the translation was externally controlled and back translated into English by a bilingual speaker of English. Discrepancies between the versions were assessed and resolved by consensus discussion. Nine items are rated on a five-point Likert scale (from 1 = *(almost) never[fast) nie]* to 5 = *(almost) always*/[*(fast) immer]*). A mean score was computed, with higher values indicating higher levels of bedtime procrastination. The scale showed excellent internal consistency in the present sample (Cronbach’s *α* = 0.93), similar to Kroese et al.’s (2014) findings (Cronbach’s α = 0.92, *N* = 177).

**Table 1:** Item characteristics for the nine-item Bedtime Procrastination Scale in its original form (English) and its German translation (BPS-G).

Item number	Item (Original)	Item (German)	*M*	*SD*	*P_i_*	*r_it_*
1	I go to bed later than I had intended.	Ich gehe später ins Bett, als ich es mir vorgenommen habe.	3.22	1.21	55.57	0.81
2	I go to bed early if I have to get up early in the morning.*	Ich gehe früh ins Bett, wenn ich am nächsten Morgen früh aufstehen muss.*	2.61	1.17	40.37	0.66
3	If it is time to turn off the lights at night I do it immediately.*	Wenn es Zeit ist, das Licht auszuschalten, tue ich dies umgehend.*	2.76	1.31	43.89	0.60
4	Often I am still doing other things when it is time to go to bed.	Häufig bin ich noch mit anderen Dingen beschäftigt, wenn es Zeit ist, ins Bett zu gehen.	3.15	1.16	53.74	0.75
5	I easily get distracted by things when I actually would like to go to bed.	Ich lasse mich leicht von Dingen ablenken, wenn ich eigentlich ins Bett gehen möchte.	2.97	1.21	49.22	0.74
6	I do not go to bed on time.	Ich gehe nicht pünktlich ins Bett.	3.04	1.24	50.64	0.84
7	I have a regular bedtime which I keep to.*	Ich habe eine regelmäßige Schlafenszeit, an die ich mich halte.*	2.97	1.32	49.36	0.69
8	I want to go to bed on time but I just do not.	Ich möchte rechtzeitig ins Bett gehen, aber ich mache es nicht.	2.81	1.24	45.16	0.81
9	I can easily stop with my activities when it is time to go to bed.*	Ich kann ganz einfach meine Tätigkeiten beenden, wenn es Zeit ist, ins Bett zu gehen.*	3.00	1.21	50.05	0.70

*Inverted item; P_i_, difficulty index; r_it_, part-whole corrected discriminatory power.

#### 2.3.2 General procrastination

To assess the broader concept of procrastination, the nine-item German short version of the General Procrastination Scale (GPS-K; Klingsieck and Fries, 2012) was applied. It was developed and validated after translation and revision of Lay’s (1986) General Procrastination Scale (GPS). Items are rated on a four-point Likert scale ranging from 1 = *extremely uncharacteristic* to 4 = *extremely characteristic*. Item scores were averaged to create a mean score for general procrastination. Internal consistency in the present sample was excellent (Cronbach’s *α* = 0.91), in accordance with Klingsieck and Fries (2012; Cronbach’s α = 0.86, *N* = 218).

#### 2.3.3 Self-control

Bertrams and Dickhäuser (2009) translated and revised Tangney et al.’s (2004) Self-Control Scale (SCS). Despite there being a 36-item version, they focused on the shorter 13-item questionnaire because of its economic use in research. Items are rated on a five-point Likert scale ranging from 1 = *not at all* to 5 = *very much*. Item scores were averaged to create a mean score, where higher scores indicate higher levels self-control. Internal consistency in the present sample was good (Cronbach’s = 0.84), comparable to Bertrams and Dickhäuser (2009; Cronbach’s = 0.79, *N* = 316).

#### 2.3.4 Affect

Affect was assessed using the PANAS-G, developed by Breyer and Bluemke (2016), adapted from Watson et al. (1988)). The questionnaire consists of 20 items describing different emotional states where ten items assess positive affect and ten assess negative affect. Items are answered on a five-point scale (ranging from 1 = *not at all* to 5 = *extremely*). The scale provides satisfactory reliability for both dimensions (Cronbach’s *α* = 0.88–0.89), similar to original findings (Breyer and Bluemke, 2016; Cronbach’s α = 0.86–0.93, *N* = 4,188).

#### 2.3.5 Big five

Rammstedt and John (2007) revised and translated the 44-item Big Five Inventory (BFI; John et al., 1991) reducing it to a ten-item scale. Each of the five personality dimensions is represented by two items, reflecting the high and low pole of each factor. Items are rated on five-point Likert scale (from 1 = *disagree strongly* to 5 *= agree strongly*). An additional item for agreeableness was added to increase validity and reliability, making it the BFI-10 + 1. Means were computed for each personality dimension. Reliability was assessed using the Spearman-Brown coefficient for two-item scales, with results ranging from 0.50 (Conscientiousness) to 0.78 (Extraversion). Cronbach’s alpha for agreeableness was 0.53. Rammstedt and John (2007) report similar findings (mean α = 0.75, *N* = 457).

#### 2.3.6 Satisfaction with life

Life satisfaction was assessed using the short scale for general life satisfaction (L-1; Beierlein et al., 2014), a single-item measure with an 11-point response scale (ranging from 0 = extremely unsatisfied to 10 = extremely satisfied). Reliability was assessed using retest data from T2 (r_tt_ = 0.48). Reliability was therefore lower than in the original sample (r_tt_ = 0.67, *N* = 335).

#### 2.3.7 Sleep quality

The German version of the Pittsburgh Sleep Quality Index was validated by Backhaus et al. in 2002 (adapted from Buysse et al., 1989). It allows investigation of differences in sleep behaviors and can recognize first signs of sleep disorders. While the questionnaire originally consists of 10 items, the present study used only four questions examining typical bed- and wake-up times as well as the amount of sleep one typically got over the last 4 weeks. Means were calculated for sleep latency and sleep duration. Retest reliability for the PSQI score was high (*r_tt_* = 0.86; Backhaus et al., 2002). As all four items represent different aspects of sleep behavior, internal consistency was not assessed in the present sample. Furthermore, two items were used to assess general sleep quality (over the last week) and short-term sleep quality (of last night) on a scale from 0 = *very bad* to 10 = *excellent*.

### 2.4 Data analysis

#### 2.4.1 Data preparation and cleaning

Data preparation and cleaning were performed using SPSS (IBM Corp, 2021; Version 29.0.2.0 (20)). At first, two variables were created to perform necessary exclusions of participants. The first variable excluded participants who did not complete the BPS. The second variable excluded participants who showed extreme completion times (more than two standard deviations from the mean of the relative completion speed). Next, a sensitivity analysis was conducted to examine whether dropout of participants at T2 was associated with important characteristics such as bedtime procrastination, general procrastination, positive and negative affect, neuroticism and conscientiousness. A Missing Value Analysis (MVA) revealed the amount of missing data per variable as well as patterns in the data. Little’s Test was performed to evaluate whether the assumption for a distribution of missing data at Missing Completely At Random (MCAR) was plausible. Next, frequencies for gender, age, profession, and subject of study were inspected.

The following step included calculating means for all relevant scales. To start, the Big Five factors of the BFI-10 + 1 scale were extracted and inspected for descriptives. Next, remaining means and reliability for bedtime procrastination, general procrastination, affect, life satisfaction, sleep quality and self-control were computed.

Bedtime and wake-up time were converted into time variables, to further calculate their means. Bedtime was then corrected for the 24-h-cycle by adding 24 h if reported bedtime occurred after midnight and before 6 a.m. A variable was created to check reported sleep duration for plausibility by calculating the time between reported bedtime and wake-up time and subtracting reported sleep latency. The same was done for sleep variables at T2. Lastly, mean, descriptives and reliability were calculated for the bedtime procrastination scale at T2.

#### 2.4.2 Factor analyses

Prior to factor analyses, BPS items were inspected for several parameters. Analyses were conducted using R Studio (Posit Team, 2025; Version 2025.05.0 + 496), namely *tidyverse* (Wickham et al., 2019), *psych* (Revelle, 2026) and *lavaan* (Rosseel, 2012). To illustrate our findings, we used an axis of 0 to 6 for histograms but retained the response scale of 1 to 5.

Before running an exploratory factor analysis, necessary pretests were applied (for an overview see Williams et al., 2010). For the confirmatory factor analysis, a model with one factor was specified first. Maximum likelihood estimation was used to assess model fit. The metric of the latent variable was determined by fixating the factor loading of the indicator variable (item one) to one. Next, the empirical and estimated covariance matrixes were inspected. Model fit was evaluated using the Comparative Fit Index (CFI; Bentler, 1990), the TLI, the RMSEA and the Standardized Root Mean Square Residual (SRMR; Bentler, 1995).

#### 2.4.3 Validity analyses

To test for construct validity (i.e., explanatory modeling), relations to similar as well as distinguishable constructs were investigated by examining Pearson correlations and their significance level between bedtime procrastination scores and all variables of interest. Latent correlations were computed to inspect true correlations of the validation constructs by using confirmatory factor analysis (see Supplementary materials). The analysis was done with the same sample as the exploratory factor analysis. To assess criterion validity (i.e., predictive modeling), correlational analysis and linear regression were applied to test if bedtime procrastination predicts outcomes like life satisfaction, sleep quality and the number of hours slept per night.

Prior to the analyses, all regression models were inspected for linearity, homoscedasticity, normality of residuals and outliers. Models indicating heteroscedasticity were corrected by estimating robust standard errors. Hierarchical models were also tested for multicollinearity. Exploratory, incremental validity was also explored using hierarchical regression models. The first model investigated whether bedtime procrastination could explain negative affect over the influence of neuroticism. Two following models assessed if bedtime procrastination could predict sleep quality over general procrastination. Calculated variances where then subtracted to obtain additional explained variance (Δ*R^2^*). Results were tested for significance using Analysis of Variance (ANOVA). Predictive validity was tested using Pearson correlations to investigate data for relations between bedtime procrastination at T1 and sleep quality as well as bedtime and wake-up time at T2 6 weeks later.

Additionally, analyses were conducted to explore the data for differences in bedtime procrastination scores regarding gender and age. Sleep outcome results were supplemented with analyses regarding typical bed- and wake-up times as well as sleep latency and sleep duration. Finally, retest reliability of the BPS-G was computed using Pearson’s *r*. No correction for multiple comparisons was applied.

## 3 Results

### 3.1 Descriptives

Little’s test revealed significant results (χ^2^ (151) = 279.627, *p* < 0.001), indicating that the data were not missing completely at random (MCAR). However, sensitivity analysis revealed no significant differences for bedtime procrastination, general procrastination, conscientiousness, neuroticism, or affect at T1 between participants who completed T2 and those who dropped out (all *p* > 0.05), suggesting that dropout was not systematically related to these variables. Inspection of patterns in the data showed an overall proportion of missing data below 5% across all variables, which represents an acceptable amount under the missing at random (MAR) assumption with low practical impact. Because of this, no imputation was applied and analyses were conducted using available data (Dettori et al., 2018; Van Buuren, 2018). Most reported sleep durations at T1 corresponded closely to calculated hours of sleep (*r* = 0.57, *p* < 0.001), the same applied for T2 (*r* = 0.63, *p* < 0.001).

### 3.2 Item characteristics

Histograms revealed no extreme values or outliers. Table 1 depicts mean, standard deviation, difficulty, and discriminatory power for each item. Minimum and maximum values indicate that the full response scale was used for all items. Item difficulties were within an acceptable range, with moderate difficulty levels across items, indicating that items were able to differentiate well between participants with high and low bedtime procrastination scores. Corrected item-total correlations suggested excellent discriminatory power for all items.

### 3.3 Factor analysis

Inspection of the correlation matrix revealed substantial relationships between items (all *r* > 0.30). Bartlett’s test was significant (χ^2^ (36) = 3364.43, *p* < 0.001), indicating that the data were suitable for EFA. Examination of the determinant of the correlation matrix revealed no issues with linear dependencies. The KMO measure indicated excellent sampling adequacy (MSA = 0.93) exceeding the recommended threshold of 0.60 (Kaiser and Rice, 1974).

**Figure 1:**
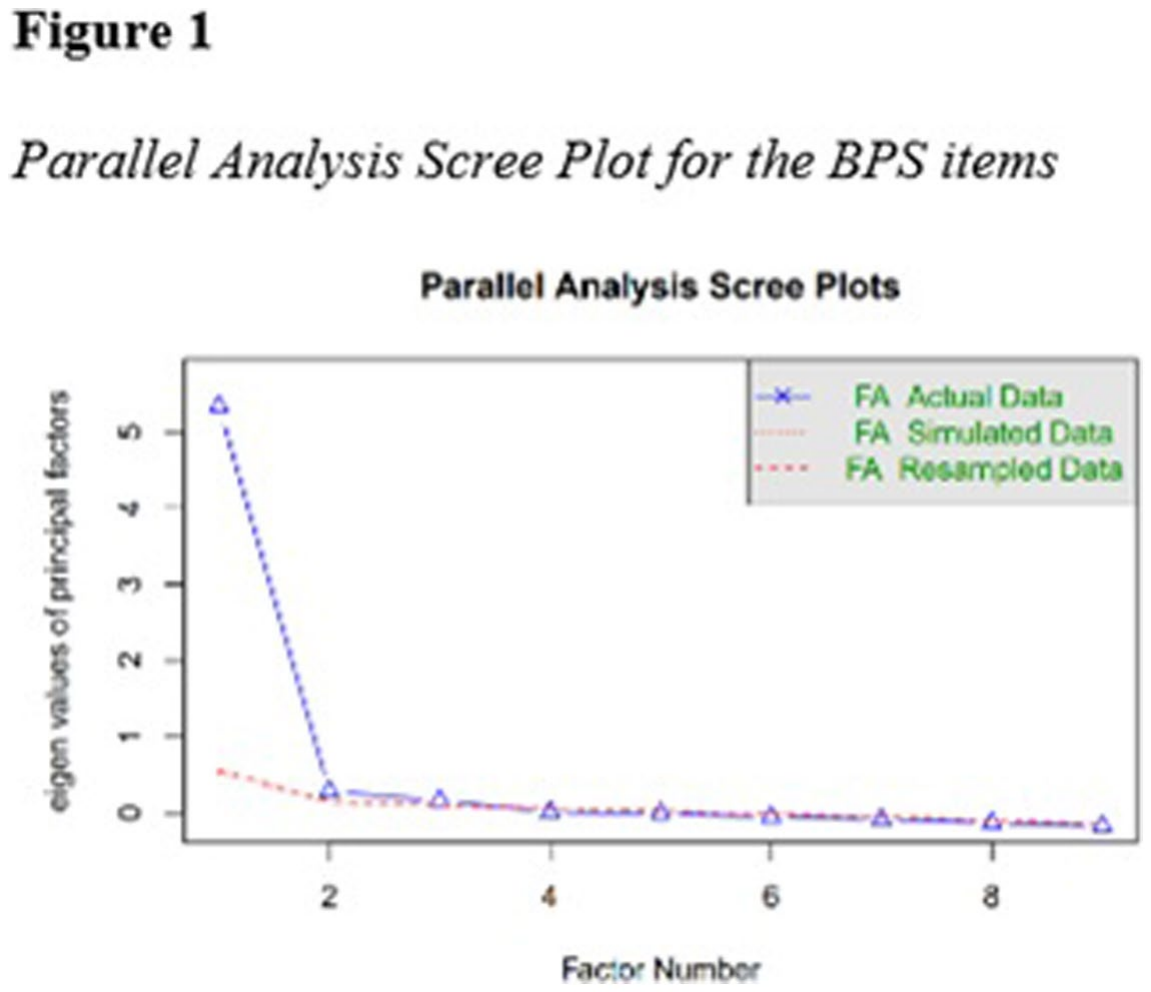
Parallel analysis scree plot for the BPS items.

Using maximum likelihood, parallel analysis and the Minimum-Average-Partial test were conducted to determine the number of factors to retain. The Scree plot in Figure 1 illustrates the results of the parallel analysis. While comparison of the sample data eigenvalues to those from simulated data suggests the presence of three factors, only the first factor showed a substantial eigenvalue (*λ* = 5.36). Furthermore, the MAP test indicated a one-factor solution, as it resulted in the lowest average squared partial correlation (0.03). Considering both results, the retention of one factor was decided. Considering this, no rotation was applied.

Table 2 shows the results of the factor analyses. EFA revealed satisfactory factor loadings (all > 0.30) while communality ranged from 0.36 to 0.81. The retained factor was interpreted as “Bedtime Procrastination” as an underlying construct, explaining 59% of total variance. A reliability analysis using McDonald’s omega (McDonald, 1999, as cited in Hayes and Coutts, 2020) indicated excellent internal consistency (ω_t_ = 0.94). Furthermore, the results of the EFA suggested that all items are suitable for measuring bedtime procrastination, which is why no further item selection was applied. Model fit showed marginally acceptable results (TLI = 0.92; RMSEA = 0.12, 90% *CI* [0.101; 0.129]).

**Table 2:** Results of the factor analyses.

Item	FL (EFA, CFA)	*h^2^*
Item 1	0.86	0.75
Item 2	0.67	0.44
Item 3	0.60	0.36
Item 4	0.78	0.61
Item 5	0.78	0.61
Item 6	0.90	0.81
Item 7	0.70	0.49
Item 8	0.86	0.74
Item 9	0.71	0.50

*N* = 548. Maximum likelihood was used for factor extraction. FL, factor loading; h^2^, communality. EFA and CFA factor loadings are identical since they are based on the same sample.

CFA was calculated following EFA to inspect model fit. Results for the model evaluation included a significant chi-squared test (χ^2^ (27) = 225.328, *p* < 0.001), indicating poor model fit. However, as the test is highly sensitive to sample size, further fit indices were inspected. While the RMSEA (0.12, 90% *CI* [0.102; 0.130]) suggested a less suitable model fit, the CFI (0.94) and the TLI (0.92) as well as the SRMR (0.04) showed sufficient fit, providing partial support for the hypothesized unidimensional structure. Modification indices revealed correlated residuals between items 4 and 5. Allowing this in the model slightly improved model fit (CFI = 0.96, TLI = 0.95, RMSEA = 0.09, SRMR = 0.04).

### 3.4 Construct validity

Table 3 displays correlations between the BPS and the measures used for validation. As expected, there was a significant moderate positive relation between bedtime procrastination and general procrastination. Participants with higher levels of bedtime procrastination tended to generally procrastinate more. Bedtime procrastination was also positively associated with negative affect, indicating that participants with higher bedtime procrastination experienced more negative affect. However, no significant correlation was found for bedtime procrastination and neuroticism (95% *CI* [−0.19, 0.15]).

**Table 3:** Descriptive statistics and Pearson correlations for bedtime procrastination and validation measures.

Variable		*M*	*SD*	*r*
	Bedtime procrastination	2.95	0.98	–
(a)	General procrastination	2.51	0.76	−0.38**
(b)	Neuroticism	3.23	0.98	−0.0700
(c)	Negative affect	2.10	0.81	0.24**
(d)	Self-control	3.13	0.65	−0.38**
(e)	Conscientiousness	3.68	0.76	−0.29**
(f)	Positive Affect	2.67	0.76	−0.21**
(g)	Openness	3.70	0.99	−0.0500
(h)	Agreeableness	3.67	0.68	−0.0100
(i)	Extraversion	3.28	0.98	−0.0300

Correlations refer to association with bedtime procrastination. **p < 0.01.

To additionally estimate latent correlations, full information maximum likelihood was used. The correlation matrix (see Supplementary materials) revealed multicollinearity between the factors self-control and conscientiousness (*r* = 0.99), resulting in a non-positive definite covariance matrix. Therefore, the findings should be interpreted with caution. Since this relation was not central to the primary research questions in this study, no further model adjustments were applied.

Significant moderate negative relations were found for bedtime procrastination and self-control as well as for conscientiousness, suggesting that higher levels of bedtime procrastination are associated with fewer self-control as well as decreased conscientiousness. Additionally, the relation between bedtime procrastination and positive affect appeared as expected: Participants with higher bedtime procrastination scores experienced lower levels of positive affect.

Coefficients for discriminant validity met expectations: No significant correlations were found for openness, agreeableness and extraversion, further supporting bedtime procrastination as a unique and independent construct.

### 3.5 Criterion validity

As shown in Table 4, bedtime procrastination showed a significant negative association with all health and sleep-related outcomes. As expected, higher levels of bedtime procrastination were associated with lower life satisfaction, with an explained variance of 2.5%. Participants reported an average general sleep quality of 6.92 (*SD* = 2.10) and an average short-term sleep quality of 7.16 (*SD* = 2.40). Higher levels of bedtime procrastination seem to be negatively linked to sleep quality in a short time span as well as long-term. Lastly, it was found that participants who reported higher bedtime procrastination tendencies showed a decreased sleep duration: On average, participants slept 7.12 h per night (*SD* = 1.09). A one-unit increase in bedtime procrastination was associated with approximately 22 min less sleep per night.

**Table 4:** Regression analyses with bedtime procrastination as predictor.

Outcome variable	*B*	*SE*	*t*	*R^2^*	*p*	*r*
Life satisfaction	−0.40	0.10	−3.61	0.02	< 0.001	−0.16
General sleep quality	−0.76	0.10	−7.36	0.09	< 0.001	−0.30
Short-term sleep quality	−0.73	0.12	−5.900	0.07	< 0.001	−0.26
Sleep duration	−0.37	0.06	−5.960	0.08	< 0.001	−0.28

B, unstandardized regression coefficients; SE, Standard Error; R^2^, coefficient of determination.

Furthermore, additional regression analyses showed that higher levels of bedtime procrastination were associated with later bedtimes at T1 [*F*(1, 531) = 53.77, *p* < 0.001]. Participants reported an average bedtime of 10:39 p.m. (*SD* = 2:52 h). Each one-unit increase in bedtime procrastination was associated with going to bed approximately 62 min later (*R^2^* = 0.09).

A similar pattern occurred for wake-up times: Higher levels of bedtime procrastination were positively associated with later wake-up times at T1 [*F*(1, 531) = 73.81, *p* < 0.001]. While participants reported an average wake-up time of 07:24 a.m. (*SD* = 1:37 h), increased bedtime procrastination was associated with getting up approximately 41 min later (*R^2^* = 0.12).

Regression analysis also revealed that bedtime procrastination was associated with the time it took participants to fall asleep at T1 [*F*(1, 531) = 14.6, *p* < 0.001]. On average, it took participants 24.97 min to fall asleep (*SD* = 28.56). A one-unit increase in bedtime procrastination accounted for additional 5.6 min of sleep onset latency.

### 3.6 Exploratory analysis

Pearson’s *r* revealed excellent retest reliability for the BPS (*r_tt_* = 0.80). Regression analysis revealed nonsignificant results for differences in bedtime procrastination between genders [*F*(1, 541) = 3.51, *p* = 0.06]. Male participants reported slightly higher levels in bedtime procrastination (*M* = 3.07, *SD* = 0.80) than females (*M* = 2.90, *SD* = 1.04). The model explained a small amount of variance (*R^2^* = 0.06). In addition, it was found that bedtime procrastination decreases with age [*F*(1, 546) = 16.78, *p* < 0.001].

#### 3.6.1 Incremental validity

Hierarchical regression demonstrated that bedtime procrastination provided significant incremental validity over neuroticism [*F*(1, 519) = 61.03, *R^2^* = 0.11, *p* < 0.001], explaining an additional 5% of variance in negative affect (Δ*R^2^* = 0.05, *p* < 0.001). Together, the predictors accounted for 15% of total variance [*F*(2, 518) = 47.1, *p* < 0.001]. The second model showed similar results. While general procrastination was connected to general sleep quality [*F*(1, 531) = 19.04, *R^2^* = 0.03, *p* < 0.001], bedtime procrastination [*F*(2, 530) = 28.14, *R^2^* = 0.09, *p* < 0.001] explains an additional 6% of variance (Δ*R^2^* = 0.06, *p* < 0.001). General procrastination was also linked to short-term sleep quality [*F*(1, 531) = 7.21, *R^2^* = 0.01, *p* = 0.01], but bedtime procrastination again added another 5% of explained variance (Δ*R^2^* = 0.05, *p* < 0.001). Both predictors accounted for a total of 6.2% of total variance [*F*(2, 530) = 18.57, *p* < 0.001]. ANOVA confirmed the increases in explained variance (all *p* < 0.001).

#### 3.6.2 Predictive validity

Correlational analyses showed weak negative associations for bedtime procrastination at T1 and general sleep quality (*r* = −0.12, 95% *CI* [−0.31; 0.09]; *p* = 0.27) as well as short-term sleep quality (*r* = −0.03, 95% *CI* [−0.23; 0.18]; *p* = 0.80) at T2 that did not reach statistical significance. Bedtime procrastination was linked to bedtime at T2 [*F*(1, 92) = 12.23, *p* < 0.001], namely a delay in bedtime of approximately 61 min (*R^2^* = 0.12). Similar results emerged for wake-up time at T2 [*F*(1, 92) = 18.91, *p* < 0.001], with higher levels of bedtime procrastination associated with getting up approximately 47 min later (*R^2^* = 0.17).

Bedtime procrastination was also connected to sleep latency at T2 [*F*(1, 91) = 4.18, *p* = 0.04], with a delay in sleep onset of approximately 8 min (*R^2^* = 0.04).

## 4 Discussion

The present study aimed to translate, culturally adapt, and psychometrically evaluate a German version (BPS-G) of the Bedtime Procrastination Scale (BPS; Kroese et al., 2014). Overall, the findings provide strong support for the reliability and validity of the instrument. The German version demonstrated excellent item characteristics, high internal consistency, good temporal stability, and meaningful associations with theoretically related constructs. Furthermore, bedtime procrastination showed expected relationships with sleep- and well-being-related outcomes, supporting the practical relevance of the construct. Taken together, the results indicate that the BPS-G is a reliable and valid instrument for assessing bedtime procrastination in German-speaking populations.

### 4.1 Factor structure and reliability

The first objective of the present study was to investigate whether the German adaptation retained the unidimensional structure proposed for the original scale (Kroese et al., 2014). Results from the exploratory factor analysis clearly supported a one-factor solution. All items displayed substantial factor loadings and satisfactory communalities, while the retained factor explained 59% of the total variance. These findings are highly consistent with previous validation studies conducted in Dutch, Spanish, Chinese, and Japanese samples (Brando-Garrido et al., 2022; Cai et al., 2022; Hazumi et al., 2024; Kroese et al., 2014), suggesting that bedtime procrastination represents a robust and coherent construct across cultural contexts. The confirmatory factor analysis provided partial support for the proposed one-factor model. While the CFI, TLI, and SRMR indicated acceptable model fit, the RMSEA exceeded conventional cut-off values. A reason for that could be residual covariance between items 4 and 5, as they share similar wording and conceptual structure which likely contributed to item redundancy. However, the elevated RMSEA should be interpreted cautiously. Methodological research has demonstrated that RMSEA may overestimate model misfit in relatively simple models with few indicators and low degrees of freedom (Kenny et al., 2015). Furthermore, researchers have increasingly cautioned against rigid adherence to universal cut-off criteria and recommend evaluating model fit based on multiple indices and substantive theory (Marsh et al., 2004). In the present study, the strong factor loadings, excellent reliability estimates, satisfactory SRMR values, and convergence with the EFA results collectively support the interpretation of a largely unidimensional construct.

Modification indices suggested correlated residuals between Items 4 and 5, both of which refer to remaining engaged in other activities when intending to go to bed. Allowing this covariance improved model fit considerably, suggesting that these items share some content-specific variance beyond the general bedtime procrastination factor. Similar residual dependencies are commonly observed in brief self-report scales containing semantically related items (Bandalos, 2021). Importantly, accounting for this covariance did not alter the substantive interpretation of the scale as measuring a single underlying construct.

The reliability findings further support the quality of the BPS-G. Internal consistency was excellent and comparable to values reported for the original version and previous adaptations (Brando-Garrido et al., 2022; Hazumi et al., 2024; Kroese et al., 2014). Additionally, the scale demonstrated excellent retest reliability over a six-week period, indicating that bedtime procrastination reflects a relatively stable individual tendency rather than merely transient fluctuations in sleep behavior. This finding is consistent with conceptualizations of bedtime procrastination as a habitual self-regulatory difficulty (Kroese et al., 2014; Nauts et al., 2019).

### 4.2 Convergent and discriminant validity

The observed pattern of correlations largely supported the hypothesized nomological network of bedtime procrastination. As expected, bedtime procrastination was moderately associated with general procrastination, indicating that individuals who delay bedtime also tend to postpone other intended activities. This finding is consistent with theoretical accounts describing procrastination as a broader self-regulation failure (Steel, 2007; Van Eerde and Klingsieck, 2018). Furthermore, bedtime procrastination showed moderate negative associations with self-control and conscientiousness. These findings closely mirror previous research identifying self-control as one of the strongest correlates of bedtime procrastination (Kadzikowska-Wrzosek, 2021; Kroese et al., 2014). Individuals with lower self-regulatory capacities may experience greater difficulties disengaging from rewarding evening activities, despite recognizing the long-term benefits of adequate sleep. The present findings therefore support the conceptualization of bedtime procrastination as a behavioral manifestation of impaired self-regulation. As expected, bedtime procrastination was also associated with higher negative affect and lower positive affect. This pattern is consistent with recent evidence suggesting that emotional states play an important role in bedtime procrastination (Tang, 2024). Individuals experiencing negative emotions may delay bedtime as a form of short-term mood repair or compensatory leisure behavior, despite potential long-term costs for sleep and well-being. Such interpretations align with emerging models that view bedtime procrastination as the result of interactions between self-regulatory difficulties and affective processes (Grabo and Bellingrath, 2026).

Contrary to expectations, no informative association was found between bedtime procrastination and neuroticism. This finding differs from some previous studies that reported small positive associations between both constructs (e.g., Carlson and Williams, 2025). One explanation may involve measurement characteristics. Neuroticism was assessed using the BFI-10 + 1, an ultra-short personality measure designed for economical assessment rather than comprehensive trait coverage, where each personality dimension is represented by only two items (Rammstedt and John, 2007). Consequently, reduced reliability and limited content breadth may have attenuated associations. Alternatively, the findings may suggest that bedtime procrastination is more strongly linked to behavioral self-regulation capacities, such as self-control and conscientiousness, than to broad dispositional emotional vulnerability. Future research employing more comprehensive personality assessments should further examine this issue. Evidence for discriminant validity was strong. As hypothesized, bedtime procrastination showed no meaningful associations with openness, agreeableness, or extraversion. This pattern supports the assumption that bedtime procrastination constitutes a specific self-regulatory phenomenon rather than a reflection of general personality functioning. Similar findings have been reported in previous validation studies (Kroese et al., 2014; Brando-Garrido et al., 2022).

### 4.3 Criterion, incremental, and predictive validity

The criterion validity analyses further demonstrated the practical significance of bedtime procrastination. Higher bedtime procrastination scores were associated with lower life satisfaction, poorer sleep quality, and shorter sleep duration. These findings closely correspond to a growing body of evidence linking bedtime procrastination to adverse sleep outcomes and reduced psychological well-being (Hill et al., 2022; Ma et al., 2022; Zhao et al., 2024). Particularly noteworthy is the finding that a one-unit increase in bedtime procrastination corresponded to approximately 22 min less sleep per night. Given that even moderate reductions in sleep duration can accumulate over time and negatively affect physical and mental health, these results underline the public health relevance of bedtime procrastination (Chaput et al., 2018). However, the findings need to be interpreted in the light of the in places low reliability of the applied measures. Importantly, bedtime procrastination demonstrated incremental validity beyond theoretically related constructs. Bedtime procrastination explained additional variance in negative affect beyond neuroticism and substantially improved the prediction of both general and short-term sleep quality beyond general procrastination. These findings strengthen the argument that bedtime procrastination represents a distinct behavioral construct rather than merely a sleep-specific manifestation of general procrastination. Similar conclusions have been drawn in recent investigations emphasizing the unique role of bedtime procrastination for sleep-related outcomes (Nauts et al., 2019; Rasouli et al., 2025). Additional analyses revealed that men reported slightly higher levels of bedtime procrastination than women. The observed effect was not statistically significant and the explained variance was small, suggesting that gender differences are of limited practical relevance. Previous findings regarding gender differences in bedtime procrastination have been inconsistent, with some studies reporting no differences and others finding slightly elevated levels among men (Nauts et al., 2019). One possible explanation is that men may engage more frequently in evening leisure activities such as gaming, media consumption, or online entertainment, which is an antecedent of delayed bedtimes (Nauts et al., 2019). However, given the small effect size, bedtime procrastination should primarily be regarded as a self-regulatory phenomenon that occurs across genders rather than a gender-specific behavioral pattern. Future research should investigate potential mechanisms underlying these differences, including evening media use, work schedules, chronotype, and self-regulatory processes. Finally, evidence for predictive validity was more nuanced. Bedtime procrastination significantly predicted later bedtimes, later wake-up times, and longer sleep latency 6 weeks later. These findings are theoretically meaningful, as they demonstrate that bedtime procrastination is associated with future behavioral sleep patterns rather than merely concurrent self-reports. However, bedtime procrastination did not significantly predict subjective sleep quality at follow-up. This discrepancy may reflect the multifactorial nature of sleep quality, which is influenced by numerous physiological, environmental, and psychological factors beyond bedtime behavior alone. Consequently, bedtime procrastination may exert stronger and more direct effects on behavioral sleep parameters than on subjective evaluations of sleep quality over extended periods.

### 4.4 Practical implications

The present findings have several implications for both research and practice. First, the BPS-G provides researchers with a psychometrically sound instrument that facilitates the investigation of bedtime procrastination in German-speaking populations and enables meaningful cross-cultural comparisons. Given the growing international interest in bedtime procrastination, the availability of validated measures across languages is essential for advancing cumulative research in this area. For clinical research and practice, it is important to study the pathophysiology of bedtime procrastination and how it might be linked to behavioral addictions (e.g., media use), sleep disorders and other conditions associated with neurobiological attention and reward imbalances (e.g., ADHD) (Becker and Langberg, 2017; Liu et al., 2025). It also deserves attention as a behavioral coping strategy, for instance, to avoid or distract from stressful situations (Huang et al., 2024). Due to its private nature (bedtime is often limited to one’s home), bedtime procrastination might be a form of private coping that is easier to target in personalized interventions as opposed to other forms of procrastination (e.g., academic) that can have secondary social benefits (e.g., procrastinating in fear of social exclusion; Kennedy and Tuckman, 2013).

Second, the observed associations with sleep quality, sleep duration, and well-being highlight bedtime procrastination as a potentially important target for intervention. Existing research suggests that interventions targeting self-regulation, implementation intentions, emotion regulation, and bedtime routines may be effective in reducing bedtime procrastination and improving sleep outcomes (Van Eerde and Klingsieck, 2018; Rasouli et al., 2025). The BPS-G may therefore serve as a useful screening and evaluation instrument in both clinical and health-promotion settings.

### 4.5 Limitations and future directions

Several limitations should be considered when interpreting the present findings. First, all variables were assessed using self-report measures. Although self-report represents the standard approach in bedtime procrastination research, shared method variance and recall biases cannot be ruled out. Future studies should complement self-report assessments with objective measures such as actigraphy, sleep diaries, or wearable sleep-tracking devices (Hickman et al., 2024).

Second, the sample was predominantly female and included a substantial proportion of younger adults. Although bedtime procrastination might be particularly relevant in these populations, the generalizability of the findings to older and more representative community samples remains to be established. Third, although attrition analyses suggested no systematic dropout effects, the longitudinal subsample was substantially smaller than the baseline sample. Future studies should replicate the predictive validity findings using larger longitudinal samples and longer follow-up periods. Fourth, measurement invariance across age and gender groups was not examined. Because small but significant gender differences emerged, future studies should additionally examine measurement invariance across gender groups to determine whether observed differences reflect true mean-level differences in bedtime procrastination or potential differences in item functioning.

Fifth, chronotype (especially late chronotype or “eveningness”) was identified as one potential factor that is proposed to significantly influence bedtime procrastination (Kühnel et al., 2018). Although not considered in the present study, it could potentially challenge the self-regulatory perspective on bedtime procrastination and therefore deserves broader examination in future studies.

Finally, although the overall evidence supported a one-factor structure, the elevated RMSEA and the observed residual covariance between conceptually related items indicate that the latent structure of bedtime procrastination may warrant further investigation. Future studies could compare alternative modeling approaches, including bifactor models, exploratory structural equation modeling (ESEM), or method-factor models that explicitly account for reverse-worded items. Such approaches may help clarify whether the observed residual variance reflects substantive facets of bedtime procrastination or methodological artifacts. Additionally, since the one-factor structure had been previously established and was therefore theoretically expected, both factor analyses were conducted on the same sample. The CFA can therefore not be interpreted as an independent confirmation of the EFA. In the future, studies could further strengthen evidence for structural validity of the BPS-G by replicating the CFA findings in independent samples.

## 5 Conclusion

The present study provides the first comprehensive psychometric evaluation of the German version (BPS-G) of the Bedtime Procrastination Scale (Kroese et al., 2014). The findings indicate that the BPS-G is a reliable, valid, and temporally stable instrument for assessing bedtime procrastination. The scale demonstrated strong internal consistency, support for a predominantly unidimensional structure, meaningful convergent and discriminant validity, incremental validity beyond related constructs, and associations with important sleep- and well-being-related outcomes. Accordingly, the BPS-G represents a valuable tool for future research on sleep-related self-regulation and may contribute to the identification of individuals at risk for maladaptive bedtime behaviors and associated sleep problems in German-speaking populations.

## Data Availability

The original contributions presented in the study are included in the article/Supplementary material, further inquiries can be directed to the corresponding author.
