# Supplementary material for: Sleepless in Germany: psychometric validation of a German version of the Bedtime Procrastination Scale
Source: Front Psychol. 2026 Sep 8;17:1918123. doi: 10.3389/fpsyg.2026.1918123 (PMC13593833; doi:10.3389/fpsyg.2026.1918123)
Supplement: Supplementary file 1 [file Table_1.DOCX]

**Appendix 1**

**Scales used in the survey and their psychometric values**

| Scale | Construct | *N* | Items | Reliability | Validity | Source |
| --- | --- | --- | --- | --- | --- | --- |
| Bedtime Procrastination Scale (BPS) | Bedtime procrastination | 177 | 9 | α = .92 | *r* = -.52 with self-regulation  *r* = .60 with general procrastination | Kroese et al. (2014) |
| General Procrastination Scale (GPS-K) | General procrastination | 218 | 9 | α = .86  *r_tt_* = .83 | *r* = .75 with API  *r* = .01 with BFI-E | Klingsieck & Fries (2012) |
| Big Five Inventory (BFI 10+1) | Big Five Dimensions | 457 | 11 | mean α = .75  mean *r_tt_* = .78 | mean *r* = .67 with NEO-PI-R | Rammstedt & John (2006) |
| Kurzskala Lebenszufriedenheit (L-1) | Life satisfaction | 539 | 1 | *r_tt_* = .67 | r = .74 with life satisfaction (multi-item)  r = -.36 with neuroticism | Beierlein et al. (2014) |
| Positive and Negative Affect Schedule (PANAS-G) | Affect | 4188 | 20 | α = .86-.93  *r_tt_* = .66 | *r* = .45 with happiness for PNP  *r* = -.37 with happiness for PNN | Breyer & Bluemke (2016) |
| Pittsburgh Sleep Quality Index (PSQI) | Sleep behaviours |  | 10 (4) | α = .85  *r_tt_* = .86 | r = -.32 with sleep onset latency | Riemann & Backhaus (1996) |
| Self-Control Scale (SCS-K-D) | Self-control | 316 | 13 | α = .79  *r_tt_* = .82 | *r* = .48 with self-regulation  *r* = -.62 with procrastination | Bertrams & Dickhäuser (2009) |
