# Supplementary material for: Sleepless in Germany: psychometric validation of a German version of the Bedtime Procrastination Scale
Source: Front Psychol. 2026 Sep 8;17:1918123. doi: 10.3389/fpsyg.2026.1918123 (PMC13593833; doi:10.3389/fpsyg.2026.1918123)
Supplement: Supplementary file 2 [file Table_2.DOCX]

**Appendix 2**

**Latent correlations of validation constructs**

| Variable | | BPS | (a) | (b) | (c) | (d) | (e) | (f) | (g) | (h) | (i) |
| --- | --- | --- | --- | --- | --- | --- | --- | --- | --- | --- | --- |
|  | Bedtime procrastination | - |  |  |  |  |  |  |  |  |  |
| (a) | General procrastination | -.43 | - |  |  |  |  |  |  |  |  |
| (b) | Neuroticism | -.10 | -.19 | - |  |  |  |  |  |  |  |
| (c) | Negative affect | -.29 | -.27 | - .40 | - |  |  |  |  |  |  |
| (d) | Self-control | -.44 | -.79 | -.33 | -.34 | - |  |  |  |  |  |
| (e) | Conscientiousness | -.40 | -.80 | -.24 | -.27 | .99 | - |  |  |  |  |
| (f) | Positive Affect | -.24 | -.29 | -.28 | -.30 | .36 | .38 | - |  |  |  |
| (g) | Openness | -.08 | -.16 | -.08 | .11 | -.07 | -.10 | .07 | - |  |  |
| (h) | Agreeableness | -.02 | .13 | -.06 | .05 | .10 | .15 | .09 | .26 | - |  |
| (i) | Extraversion | -.04 | -.02 | -.30 | -.08 | .06 | .12 | .18 | .22 | .29 | - |

*Note.* Correlations were computed using Full Information Maximum Likelihood.
