# Supplementary material for: Sleepless in Germany: psychometric validation of a German version of the Bedtime Procrastination Scale
Source: Front Psychol. 2026 Sep 8;17:1918123. doi: 10.3389/fpsyg.2026.1918123 (PMC13593833; doi:10.3389/fpsyg.2026.1918123)
Supplement: Supplementary file 3 [file Table_4.DOCX]

---

title: "BPS Analyses"

author: "Antonia Winkler"

date: "2026-05-26"

output:

pdf_document: default

html_document: default

---

\# Load packages

```{r, message = FALSE, warning = FALSE}

library(tidyverse)

library(ggplot2)

library(gridExtra)

library(readxl)

library(psych)

library(readr)

library(dplyr)

library(performance)

library(GPArotation)

library(lavaan)

library(lmtest)

library(sandwich)

library(car)

```

\# Load data

```{r, message = FALSE, warning = FALSE}

data_full <- read_csv2("BPS_data_full.csv")

data_full[data_full == -999] <- NA

data_BPS <- read_csv2("BPS_data.csv")

```

\# Histograms - item distribution

```{r, echo = FALSE, warning = FALSE}

p1 <- ggplot(data_BPS, aes(x = BP01_01)) + geom_histogram(binwidth = 1,

fill = "skyblue", color = "white") + xlim(0,6)

p2 <- ggplot(data_BPS, aes(x = BP01_02)) + geom_histogram(binwidth = 1,

fill = "skyblue", color = "white") + xlim(0,6)

p3 <- ggplot(data_BPS, aes(x = BP01_03)) + geom_histogram(binwidth = 1,

fill = "skyblue", color = "white") + xlim(0,6)

p4 <- ggplot(data_BPS, aes(x = BP01_04)) + geom_histogram(binwidth = 1,

fill = "skyblue", color = "white") + xlim(0,6)

p5 <- ggplot(data_BPS, aes(x = BP01_05)) + geom_histogram(binwidth = 1,

fill = "skyblue", color = "white") + xlim(0,6)

p6 <- ggplot(data_BPS, aes(x = BP01_06)) + geom_histogram(binwidth = 1,

fill = "skyblue", color = "white") + xlim(0,6)

p7 <- ggplot(data_BPS, aes(x = BP01_07)) + geom_histogram(binwidth = 1,

fill = "skyblue", color = "white") + xlim(0,6)

p8 <- ggplot(data_BPS, aes(x = BP01_08)) + geom_histogram(binwidth = 1,

fill = "skyblue", color = "white") + xlim(0,6)

p9 <- ggplot(data_BPS, aes(x = BP01_09)) + geom_histogram(binwidth = 1,

fill = "skyblue", color = "white") + xlim(0,6)

grid.arrange(p1, p2, p3,

p4, p5, p6,

p7, p8, p9,

ncol = 3)

```

\# Item characteristics

```{r}

psych::describe(data_BPS)

```

*item difficulty*

```{r}

difficulty <- function(x, n, max, min) {

p <- sum(x-min, na.rm = T)/(n*(max-min))

p <- p*100

p

}

difficulty(data_BPS$BP01_01, 533, 5, 1)

difficulty(data_BPS$BP01_02, 533, 5, 1)

difficulty(data_BPS$BP01_03, 533, 5, 1)

difficulty(data_BPS$BP01_04, 533, 5, 1)

difficulty(data_BPS$BP01_05, 533, 5, 1)

difficulty(data_BPS$BP01_06, 533, 5, 1)

difficulty(data_BPS$BP01_07, 533, 5, 1)

difficulty(data_BPS$BP01_08, 533, 5, 1)

difficulty(data_BPS$BP01_09, 533, 5, 1)

```

*discriminatory power*

```{r}

psych::alpha(data_BPS)

```

*correlation matrix*

```{r}

cor <- cor.plot(data_BPS)

```

\# Exploratory Factor Analysis

**Pretests**

*Bartlett's test*

```{r}

cortest.bartlett(data_BPS)

```

*Linear dependencies*

```{r}

cor_table = cor(data_BPS)

cor_table

det(cor_table)

det(cor_table) > .00001

```

*Kaiser-Meyer-Olkin test*

```{r}

KMO(data_BPS)

```

**Parallel Analysis**

```{r}

fa.parallel(data_BPS, fm = "ml", fa = "fa")

```

**Minimum-Average-Partial test**

```{r, fig.show='hide'}

vss(data_BPS)

```

**EFA**

```{r}

exfa = fa(data_BPS, nfactors = 1, fm = "ml", rotate = "none")

print.psych(exfa, cut = 0.3)

```

**Reliability**

```{r, fig.show='hide', warning=FALSE}

omega(data_BPS)

```

\# Confirmatory Factor Analysis

**Model specification**

```{r}

model = "

BP =~ BP01_01 + BP01_02 + BP01_03 + BP01_04 + BP01_05 +

BP01_06 + BP01_07 + BP01_08 + BP01_09

"

```

**Model estimation**

```{r}

fit = cfa(model, data = data_BPS, estimator = "ML")

lavInspect(fit, what = "sampstat")

lavInspect(fit, what = "implied")

lavInspect(fit, what = "resid")

resid(fit, type = "standardized")

```

**Model evaluation**

```{r}

summary(fit, fit.measures = TRUE, standardized = TRUE, rsquare = TRUE)

```

**Model indices**

```{r}

modindices(fit, sort = TRUE)

model = "

BP =~ BP01_01 + BP01_02 + BP01_03 + BP01_04 + BP01_05 + BP01_06 + BP01_07 + BP01_08 + BP01_09

BP01_04 ~~ BP01_05

"

fit2 = cfa(model, data = data_BPS, estimator = "ML")

summary(fit2, fit.measures = T, standardized = T)

```

\# Construct validity

**Convergent & Discriminant validity**

```{r}

select(data_full, c(BPS_mean, GPS_mean, PNN_mean, PNP_mean, extra_mean,

neuro_mean, open_mean, agree_mean, con_mean, SC_mean)) %>% cor.plot()

```

**Latent correlations**

```{r}

val <- "BPS =~ BP01_01 + BP01_02 + BP01_03 + BP01_04 + BP01_05 +

BP01_06 + BP01_07 + BP01_08 + BP01_09

GPS =~ GP02_01 + GP02_02 + GP02_03 + GP02_04 + GP02_05 + GP02_06 +

GP02_07 + GP02_08 + GP02_09

neuro =~ BK02_09 + BK02_19

PNN =~ PN03_02 + PN03_05 + PN03_07 + PN03_08 + PN03_09 + PN03_12 +

PN03_14 + PN03_16 + PN03_19 + PN03_20

SC =~ SR01_01 + SR01_02 + SR01_03 + SR01_04 + SR01_05 + SR01_06_recoded +

SR01_07 + SR01_08 + SR01_09 + SR01_10 + SR01_11 + SR01_12 + SR01_13

con =~ BK02_08 + BK02_03

PNP =~ PN03_01 + PN03_03 + PN03_04 + PN03_06 + PN03_10 + PN03_11 +

PN03_13 + PN03_15 + PN03_17 + PN03_18

open =~ BK02_21 + BK02_15

agree =~ BK02_07 + BK02_02 + BK02_22

extra =~ BK02_01 + BK02_16

"

fit_val = cfa(val, data = data_full, missing = "fiml")

summary(fit_val, fit.measures = TRUE, standardized = TRUE, rsquare = TRUE)

lavInspect(fit_val, "cor.lv")

```

\# Criterion validity

*life satisfaction*

```{r}

cor.test(data_full$LZ01_01, data_full$BPS_mean)

lm1 = lm(LZ01_01 ~ BPS_mean, data = data_full)

plot(lm1)

check_heteroscedasticity(lm1)

check_outliers(lm1)

summary(lm1)

```

*general sleep quality*

```{r}

cor.test(data_full$SQ01_01, data_full$BPS_mean)

lm2 = lm(SQ01_01 ~ BPS_mean, data = data_full)

plot(lm2)

check_heteroscedasticity(lm2)

check_outliers(lm2)

summary(lm2)

```

*short-term sleep quality*

```{r}

cor.test(data_full$SQ02_01, data_full$BPS_mean)

lm3 = lm(SQ02_01 ~ BPS_mean, data = data_full)

plot(lm3)

check_heteroscedasticity(lm3)

coeftest(lm3, vcov = vcovHC(lm3, type = "HC3"))

check_outliers(lm3)

summary(lm3)

```

*sleep duration*

```{r}

cor.test(data_full$SQ04_04, data_full$BPS_mean)

lm4 = lm(SQ04_04 ~ BPS_mean, data = data_full)

plot(lm4)

check_heteroscedasticity(lm4)

coeftest(lm4, vcov = vcovHC(lm4, type = "HC3"))

check_outliers(lm4)

summary(lm4)

```

**Incremental validity**

*Negative affect*

```{r}

lm5 = lm(PNN_mean ~ neuro_mean, data = data_full)

lm6 = lm(PNN_mean ~ neuro_mean + BPS_mean, data = data_full)

plot(lm6)

check_heteroscedasticity(lm6)

coeftest(lm6, vcov = vcovHC(lm6, type = "HC3"))

check_outliers(lm6)

vif(lm6)

summary(lm5)

summary(lm6)

summary(lm6)$r.squared - summary(lm5)$r.squared

anova(lm5, lm6)

```

*Sleep quality*

```{r}

lm7 = lm(SQ01_01 ~ GPS_mean, data = data_full)

lm8 = lm(SQ01_01 ~ GPS_mean + BPS_mean, data = data_full)

plot(lm8)

check_heteroscedasticity(lm8)

check_outliers(lm8)

vif(lm8)

summary(lm7)

summary(lm8)

summary(lm8)$r.squared - summary(lm7)$r.squared

anova(lm7, lm8)

lm9 = lm(SQ02_01 ~ GPS_mean, data = data_full)

lm10 = lm(SQ02_01 ~ GPS_mean + BPS_mean, data = data_full)

plot(lm10)

check_heteroscedasticity(lm10)

coeftest(lm10, vcov = vcovHC(lm10, type = "HC3"))

check_outliers(lm10)

vif(lm10)

summary(lm9)

summary(lm10)

summary(lm10)$r.squared - summary(lm9)$r.squared

anova(lm9, lm10)

```

**Predicitive validity**

```{r}

cor.test(data_full$BPS_mean, data_full$SQ01_01_T2)

cor.test(data_full$BPS_mean, data_full$SQ02_01_T2)

lm11 = lm(SQ04_02_T2 ~ BPS_mean, data = data_full)

plot(lm11)

check_heteroscedasticity(lm11)

coeftest(lm11, vcov = vcovHC(lm11, type = "HC3"))

summary(lm11)

```

\# Additional analyses

*Retest reliability*

```{r}

cor(data_full$BPS_mean, data_full$BPS_T2_mean, method = "pearson",

use = "pairwise.complete.obs")

```

*Gender differences*

```{r}

table(data_full$SD01)

data_f_m <- data_full %>% filter(SD01 != 3)

data_f_m %>% group_by(SD01) %>% summarize(BPS_gender_mean = mean(BPS_mean),

BPS_gender_sd = sd(BPS_mean))

data_full$SD01 <- factor(data_full$SD01)

leveneTest(BPS_mean ~ SD01, data = data_full)

lm12 = lm(BPS_mean ~ SD01, data = data_f_m)

check_heteroscedasticity(lm12)

coeftest(lm12, vcov = vcovHC(lm12, type = "HC3"))

check_outliers(lm12)

summary(lm12)

```

*Age differences*

```{r}

lm13 = lm(BPS_mean ~ SD02, data = data_full)

plot(lm13)

check_heteroscedasticity(lm13)

coeftest(lm13, vcov = vcovHC(lm13, type = "HC3"))

check_outliers(lm13)

summary(lm13)

```

*Sleep latency*

```{r}

lm14 = lm(SQ04_02 ~ BPS_mean, data = data_full)

plot(lm14)

check_heteroscedasticity(lm14)

coeftest(lm14, vcov = vcovHC(lm14, type = "HC3"))

summary(lm13)

```
