# Supplementary material for: Sleepless in Germany: psychometric validation of a German version of the Bedtime Procrastination Scale
Source: Front Psychol. 2026 Sep 8;17:1918123. doi: 10.3389/fpsyg.2026.1918123 (PMC13593833; doi:10.3389/fpsyg.2026.1918123)
Supplement: Supplementary file 4 [file Table_5.DOCX]

* Encoding: UTF-8.

*Data cleaning and preparation.

*Exclusion: participants with extreme completion times and no answers for the BPS.

DESCRIPTIVES VARIABLES=TIME_RSI

/STATISTICS=MEAN STDDEV MIN MAX.

*Mean = 1.066099.

*SD = 0.3419188.

*Compute variable that excludes extreme times.

COMPUTE filter_rsi = (TIME_RSI >= (1.066099 - 2*0.3419188)) AND (TIME_RSI <= (1.066099 + 2*0.3419188)).

VARIABLE LABELS filter_rsi 'Filter: TIME_RSI innerhalb �2 SD'.

VALUE LABELS filter_rsi 0 'ausgeschlossent' 1 'inkludiert'.

EXECUTE.

SELECT IF (filter_rsi = 1 AND NVALID (BP01_01 to BP01_09) = 9).

EXECUTE.

*Sensitivity analysis.

COMPUTE dropout_T2=STATUS_T2 ~= "complete" .

EXECUTE.

T-TEST GROUPS=dropout_T2(0 1)

/VARIABLES=BPS_mean GPS_mean PNN_mean PNP_mean neuro_mean con_mean

/MISSING=ANALYSIS.

*Demographics for T1 & N for T2.

COMPUTE SD02_age = SD02 +15.

EXECUTE.

FREQUENCIES VARIABLES=SD01 SD02_age SD12 SD12_07 STUDY

/STATISTICS=STDDEV MEAN

/ORDER=ANALYSIS.

FREQUENCIES VARIABLES=STATUS_T2

/ORDER=ANALYSIS.

*################################################################.

*Missing Value Analysis.

MVA VARIABLES=BK02_01 BK02_02 BK02_03 BK02_07 BK02_08 BK02_09 BK02_15 BK02_16 BK02_19 BK02_21

BK02_22 BP01_01 BP01_02 BP01_03 BP01_04 BP01_05 BP01_06 BP01_07 BP01_08 BP01_09 GP02_01

GP02_02 GP02_03 GP02_04 GP02_05 GP02_06 GP02_07 GP02_08 GP02_09 LZ01_01 PN03_01 PN03_02 PN03_03

PN03_04 PN03_05 PN03_06 PN03_07 PN03_08 PN03_09 PN03_10 PN03_11 PN03_12 PN03_13 PN03_14 PN03_15

PN03_16 PN03_17 PN03_18 PN03_19 PN03_20 SQ01_01 SQ02_01 SQ04_02 SQ04_04 SR01_01 SR01_02 SR01_03 SR01_04 SR01_05

SR01_06_recoded SR01_07 SR01_08 SR01_09 SR01_10 SR01_11 SR01_12 SR01_13

/MAXCAT=100

/CATEGORICAL= SQ04_01 SQ04_03.

*Patterns of missing data.

MULTIPLE IMPUTATION BK02_01 BK02_02 BK02_03 BK02_07 BK02_08 BK02_09 BK02_15 BK02_16 BK02_19 BK02_21

BK02_22 BP01_01 BP01_02 BP01_03 BP01_04 BP01_05 BP01_06 BP01_07 BP01_08 BP01_09 GP02_01

GP02_02 GP02_03 GP02_04 GP02_05 GP02_06 GP02_07 GP02_08 GP02_09 LZ01_01 PN03_01 PN03_02 PN03_03

PN03_04 PN03_05 PN03_06 PN03_07 PN03_08 PN03_09 PN03_10 PN03_11 PN03_12 PN03_13 PN03_14 PN03_15

PN03_16 PN03_17 PN03_18 PN03_19 PN03_20 SQ01_01 SQ02_01 SQ04_01 SQ04_02 SQ04_03 SQ04_04 SR01_01 SR01_02 SR01_03 SR01_04 SR01_05

SR01_06_recoded SR01_07 SR01_08 SR01_09 SR01_10 SR01_11 SR01_12 SR01_13

/IMPUTE METHOD=NONE

/MISSINGSUMMARIES OVERALL VARIABLES (MAXVARS=100 MINPCTMISSING=10) PATTERNS.

*Little's Test auf MCAR.

MVA VARIABLES=BK02_01 BK02_02 BK02_03 BK02_07 BK02_08 BK02_09 BK02_15 BK02_16 BK02_19 BK02_21

BK02_22 BP01_01 BP01_02 BP01_03 BP01_04 BP01_05 BP01_06 BP01_07 BP01_08 BP01_09 GP02_01

GP02_02 GP02_03 GP02_04 GP02_05 GP02_06 GP02_07 GP02_08 GP02_09 LZ01_01 PN03_01 PN03_02 PN03_03

PN03_04 PN03_05 PN03_06 PN03_07 PN03_08 PN03_09 PN03_10 PN03_11 PN03_12 PN03_13 PN03_14 PN03_15

PN03_16 PN03_17 PN03_18 PN03_19 PN03_20 SQ01_01 SQ02_01 SQ04_02 SQ04_04 SR01_01 SR01_02 SR01_03 SR01_04 SR01_05

SR01_06_recoded SR01_07 SR01_08 SR01_09 SR01_10 SR01_11 SR01_12 SR01_13

/MAXCAT=100

/CATEGORICAL=SQ04_01 SQ04_03

/EM(TOLERANCE=0.001 CONVERGENCE=0.0001 ITERATIONS=25).

*##############################################################.

*Compute Big Five variables and show descriptives and reliability.

*Extraversion.

COMPUTE extra_mean = MEAN(BK02_01, BK02_16).

EXECUTE.

DESCRIPTIVES VARIABLES=extra_mean

/STATISTICS=MEAN STDDEV MIN MAX KURTOSIS SKEWNESS.

RELIABILITY

/VARIABLES=BK02_01 BK02_16

/SCALE('ALL VARIABLES') ALL

/MODEL=SPLIT.

*Agreeableness.

COMPUTE agree_mean = MEAN(BK02_07, BK02_02, BK02_22).

EXECUTE.

DESCRIPTIVES VARIABLES=agree_mean

/STATISTICS=MEAN STDDEV MIN MAX KURTOSIS SKEWNESS.

RELIABILITY

/VARIABLES=BK02_02 BK02_07 BK02_22

/SCALE('ALL VARIABLES') ALL

/MODEL=ALPHA.

*Conscientiousness.

COMPUTE con_mean = MEAN(BK02_08, BK02_03).

EXECUTE.

DESCRIPTIVES VARIABLES=con_mean

/STATISTICS=MEAN STDDEV MIN MAX KURTOSIS SKEWNESS.

RELIABILITY

/VARIABLES=BK02_03 BK02_08

/SCALE('ALL VARIABLES') ALL

/MODEL=SPLIT.

*Neuroticism.

COMPUTE neuro_mean = MEAN(BK02_09, BK02_19).

EXECUTE.

DESCRIPTIVES VARIABLES=neuro_mean

/STATISTICS=MEAN STDDEV MIN MAX KURTOSIS SKEWNESS.

RELIABILITY

/VARIABLES=BK02_09 BK02_19

/SCALE('ALL VARIABLES') ALL

/MODEL=SPLIT.

*Openness.

COMPUTE open_mean = MEAN(BK02_21, BK02_15).

EXECUTE.

DESCRIPTIVES VARIABLES=open_mean

/STATISTICS=MEAN STDDEV MIN MAX KURTOSIS SKEWNESS.

RELIABILITY

/VARIABLES=BK02_21 BK02_15

/SCALE('ALL VARIABLES') ALL

/MODEL=SPLIT.

*Compute scale means and show descriptves and reliability.

*BPS.

COMPUTE BPS_mean=MEAN (BP01_01, BP01_02, BP01_03, BP01_04, BP01_05, BP01_06, BP01_07, BP01_08, BP01_09).

EXECUTE.

DESCRIPTIVES VARIABLES=BPS_mean

/STATISTICS=MEAN STDDEV MIN MAX KURTOSIS SKEWNESS.

RELIABILITY

/VARIABLES=BP01_01 BP01_02 BP01_03 BP01_04 BP01_05 BP01_06 BP01_07 BP01_08 BP01_09

/SCALE('ALL VARIABLES') ALL

/MODEL=ALPHA

/STATISTICS=DESCRIPTIVE CORRELATIONS

/SUMMARY=TOTAL.

*GPS.

COMPUTE GPS_mean=MEAN (GP02_01, GP02_02, GP02_03, GP02_04, GP02_05, GP02_06, GP02_07, GP02_08, GP02_09).

EXECUTE.

DESCRIPTIVES VARIABLES=GPS_mean

/STATISTICS=MEAN STDDEV MIN MAX KURTOSIS SKEWNESS.

RELIABILITY

/VARIABLES=GP02_01 GP02_02 GP02_03 GP02_04 GP02_05 GP02_06 GP02_07 GP02_08 GP02_09

/SCALE('ALL VARIABLES') ALL

/MODEL=ALPHA

/STATISTICS=DESCRIPTIVE CORRELATIONS

/SUMMARY=TOTAL.

*Life satisfaction.

DESCRIPTIVES VARIABLES=LZ01_01

/STATISTICS=MEAN STDDEV MIN MAX KURTOSIS SKEWNESS.

DESCRIPTIVES VARIABLES=LZ01_01_T2

/STATISTICS=MEAN STDDEV MIN MAX KURTOSIS SKEWNESS.

CORRELATIONS

/VARIABLES=LZ01_01 LZ01_01_T2

/PRINT=TWOTAIL NOSIG FULL

/MISSING=PAIRWISE.

*PANAS - positive affect.

COMPUTE PNP_mean=MEAN (PN03_01, PN03_03, PN03_04, PN03_06, PN03_10, PN03_11, PN03_13, PN03_15, PN03_17, PN03_18).

EXECUTE.

DESCRIPTIVES VARIABLES=PNP_mean

/STATISTICS=MEAN STDDEV MIN MAX KURTOSIS SKEWNESS.

RELIABILITY

/VARIABLES=PN03_01, PN03_03, PN03_04, PN03_06, PN03_10, PN03_11, PN03_13, PN03_15, PN03_17, PN03_18

/SCALE('ALL VARIABLES') ALL

/MODEL=ALPHA

/STATISTICS=DESCRIPTIVE CORRELATIONS

/SUMMARY=TOTAL.

*PANAS - negative affect.

COMPUTE PNN_mean=MEAN (PN03_02, PN03_05, PN03_07, PN03_08, PN03_09, PN03_12, PN03_14, PN03_16, PN03_19, PN03_20).

EXECUTE.

DESCRIPTIVES VARIABLES=PNN_mean

/STATISTICS=MEAN STDDEV MIN MAX KURTOSIS SKEWNESS.

RELIABILITY

/VARIABLES=PN03_02, PN03_05, PN03_07, PN03_08, PN03_09, PN03_12, PN03_14, PN03_16, PN03_19, PN03_20

/SCALE('ALL VARIABLES') ALL

/MODEL=ALPHA

/STATISTICS=DESCRIPTIVE CORRELATIONS

/SUMMARY=TOTAL.

*Bedtime.

* Date and Time Wizard: Convert string into time variable.

COMPUTE SQ04_01_time=number(SQ04_01, TIME5).

VARIABLE LABELS SQ04_01_time 'Anzahl geschlafener Stunden: Wann sind Sie w�hrend der letzten '+

'vier Wochen gew�hnlich abends zu Bett gegangen? ... �bliche Uhrzeit'.

VARIABLE LEVEL SQ04_01_time (SCALE).

FORMATS SQ04_01_time (TIME5).

VARIABLE WIDTH SQ04_01_time(5).

EXECUTE.

*Correction for circularity: Bedtime after midnight + 24h.

IF (SQ04_01_time < TIME.HMS(6,0,0)) SQ04_01_time = SQ04_01_time + TIME.HMS(24,0,0).

EXECUTE.

DESCRIPTIVES VARIABLES=SQ04_01_time

/STATISTICS=MEAN STDDEV MIN MAX.

*Bedtime T1.

REGRESSION

/MISSING LISTWISE

/STATISTICS COEFF OUTS R ANOVA

/CRITERIA=PIN(.05) POUT(.10) TOLERANCE(.0001)

/NOORIGIN

/DEPENDENT SQ04_01_time

/METHOD=ENTER BPS_mean.

*Wake-up time.

*Date and Time Wizard: Convert string into time variable.

COMPUTE SQ04_03_time=number(SQ04_03, TIME5).

VARIABLE LABELS SQ04_03_time 'Anzahl geschlafener Stunden: Wann sind Sie w�hrend der letzten '+

'vier Wochen gew�hnlich morgens aufgestanden? ... �bliche Uhrzeit'.

VARIABLE LEVEL SQ04_03_time (SCALE).

FORMATS SQ04_03_time (TIME5).

VARIABLE WIDTH SQ04_03_time(5).

EXECUTE.

DESCRIPTIVES VARIABLES=SQ04_03_time

/STATISTICS=MEAN STDDEV MIN MAX.

*Wake-up time at T1.

REGRESSION

/MISSING LISTWISE

/STATISTICS COEFF OUTS R ANOVA

/CRITERIA=PIN(.05) POUT(.10) TOLERANCE(.0001)

/NOORIGIN

/DEPENDENT SQ04_03_time

/METHOD=ENTER BPS_mean.

*Sleep quality.

DESCRIPTIVES VARIABLES=SQ01_01

/STATISTICS=MEAN STDDEV MIN MAX.

DESCRIPTIVES VARIABLES=SQ02_01

/STATISTICS=MEAN STDDEV MIN MAX.

*Sleep latency.

DESCRIPTIVES VARIABLES=SQ04_02

/STATISTICS=MEAN STDDEV MIN MAX.

*Sleep duration.

DESCRIPTIVES VARIABLES=SQ04_04

/STATISTICS=MEAN STDDEV MIN MAX.

*Reported sleep duration = real sleep duration?.

COMPUTE bedtime_sec = XDATE.TIME(SQ04_01_time).

COMPUTE wake_up_sec = XDATE.TIME(SQ04_03_time).

IF bedtime_sec > wake_up_sec bedtime_sec = bedtime_sec - 86400.

EXECUTE.

COMPUTE sleep_sec = (wake_up_sec - bedtime_sec) - (SQ04_02 * 60).

EXECUTE.

COMPUTE sleep_duration_calc = sleep_sec / 3600.

EXECUTE.

IF sleep_duration_calc > 14 sleep_duration_calc = $SYSMIS.

EXECUTE.

DESCRIPTIVES VARIABLES=sleep_duration_calc

/STATISTICS=MEAN STDDEV MIN MAX.

CORRELATIONS

/VARIABLES=SQ04_04 sleep_duration_calc

/PRINT=TWOTAIL NOSIG FULL

/MISSING=PAIRWISE.

*Self-control.

RECODE SR01_06 (5=1) (4=2) (3=3) (2=4) (1=5) (-999=SYSMIS) INTO SR01_06_recoded.

VARIABLE LABELS SR01_06_recoded 'SR01_06_recoded'.

EXECUTE.

COMPUTE SC_mean = MEAN(SR01_01, SR01_02, SR01_03, SR01_04, SR01_05, SR01_06_recoded, SR01_07, SR01_08, SR01_09, SR01_10, SR01_11, SR01_12, SR01_13).

EXECUTE.

DESCRIPTIVES VARIABLES=SC_mean

/STATISTICS=MEAN STDDEV MIN MAX KURTOSIS SKEWNESS.

RELIABILITY

/VARIABLES=SR01_01 SR01_02 SR01_03 SR01_04 SR01_05 SR01_06_recoded SR01_07 SR01_08 SR01_09 SR01_10

SR01_11 SR01_12 SR01_13

/SCALE('ALL VARIABLES') ALL

/MODEL=ALPHA

/STATISTICS=DESCRIPTIVE CORRELATIONS

/SUMMARY=TOTAL.

*Correlations BPS with related validation measures.

CORRELATIONS

/VARIABLES=BPS_mean GPS_mean neuro_mean PNN_mean SC_mean con_mean PNP_mean open_mean agree_mean

extra_mean

/PRINT=TWOTAIL NOSIG FULL

/MISSING=PAIRWISE.

*####################################################.

*Second measurement point (T2).

*Missing Value Analysis.

MVA VARIABLES=BK02_01_T2 BK02_02_T2 BK02_03_T2 BK02_07_T2 BK02_08_T2 BK02_09_T2 BK02_15_T2 BK02_16_T2 BK02_19_T2 BK02_21_T2

BK02_22_T2 BP01_01_T2 BP01_02_T2 BP01_03_T2 BP01_04_T2 BP01_05_T2 BP01_06_T2 BP01_07_T2 BP01_08_T2 BP01_09_T2 GP02_01_T2

GP02_02_T2 GP02_03_T2 GP02_04_T2 GP02_05_T2 GP02_06_T2 GP02_07_T2 GP02_08_T2 GP02_09_T2 LZ01_01_T2 PN03_01_T2 PN03_02_T2 PN03_03_T2

PN03_04_T2 PN03_05_T2 PN03_06_T2 PN03_07_T2 PN03_08_T2 PN03_09_T2 PN03_10_T2 PN03_11_T2 PN03_12_T2 PN03_13_T2 PN03_14_T2 PN03_15_T2

PN03_16_T2 PN03_17_T2 PN03_18_T2 PN03_19_T2 PN03_20_T2 SQ01_01_T2 SQ02_01_T2 SQ04_02_T2 SQ04_04_T2 SR01_01_T2 SR01_02_T2 SR01_03_T2 SR01_04_T2 SR01_05_T2

SR01_06_T2 SR01_07_T2 SR01_08_T2 SR01_09_T2 SR01_10_T2 SR01_11_T2 SR01_12_T2 SR01_13_T2

/MAXCAT=100

/CATEGORICAL= SQ04_01_T2 SQ04_03_T2.

*Patterns of missing data.

MULTIPLE IMPUTATION BK02_01_T2 BK02_02_T2 BK02_03_T2 BK02_07_T2 BK02_08_T2 BK02_09_T2 BK02_15_T2 BK02_16_T2 BK02_19_T2 BK02_21_T2

BK02_22_T2 BP01_01_T2 BP01_02_T2 BP01_03_T2 BP01_04_T2 BP01_05_T2 BP01_06_T2 BP01_07_T2 BP01_08_T2 BP01_09_T2 GP02_01_T2

GP02_02_T2 GP02_03_T2 GP02_04_T2 GP02_05_T2 GP02_06_T2 GP02_07_T2 GP02_08_T2 GP02_09_T2 LZ01_01_T2 PN03_01_T2 PN03_02_T2 PN03_03_T2

PN03_04_T2 PN03_05_T2 PN03_06_T2 PN03_07_T2 PN03_08_T2 PN03_09_T2 PN03_10_T2 PN03_11_T2 PN03_12_T2 PN03_13_T2 PN03_14_T2 PN03_15_T2

PN03_16_T2 PN03_17_T2 PN03_18_T2 PN03_19_T2 PN03_20_T2 SQ01_01_T2 SQ02_01_T2 SQ04_01_T2 SQ04_02_T2 SQ04_03_T2 SQ04_04_T2 SR01_01_T2 SR01_02_T2 SR01_03_T2 SR01_04_T2 SR01_05_T2

SR01_06_T2 SR01_07_T2 SR01_08_T2 SR01_09_T2 SR01_10_T2 SR01_11_T2 SR01_12_T2 SR01_13_T2

/IMPUTE METHOD=NONE

/MISSINGSUMMARIES OVERALL VARIABLES (MAXVARS=100 MINPCTMISSING=10) PATTERNS.

*Little's Test auf MCAR.

MVA VARIABLES=BK02_01_T2 BK02_02_T2 BK02_03_T2 BK02_07_T2 BK02_08_T2 BK02_09_T2 BK02_15_T2 BK02_16_T2 BK02_19_T2 BK02_21_T2

BK02_22_T2 BP01_01_T2 BP01_02_T2 BP01_03_T2 BP01_04_T2 BP01_05_T2 BP01_06_T2 BP01_07_T2 BP01_08_T2 BP01_09_T2 GP02_01_T2

GP02_02_T2 GP02_03_T2 GP02_04_T2 GP02_05_T2 GP02_06_T2 GP02_07_T2 GP02_08_T2 GP02_09_T2 LZ01_01_T2 PN03_01_T2 PN03_02_T2 PN03_03_T2

PN03_04_T2 PN03_05_T2 PN03_06_T2 PN03_07_T2 PN03_08_T2 PN03_09_T2 PN03_10_T2 PN03_11_T2 PN03_12_T2 PN03_13_T2 PN03_14_T2 PN03_15_T2

PN03_16_T2 PN03_17_T2 PN03_18_T2 PN03_19_T2 PN03_20_T2 SQ01_01_T2 SQ02_01_T2 SQ04_02_T2 SQ04_04_T2 SR01_01_T2 SR01_02_T2 SR01_03_T2 SR01_04_T2 SR01_05_T2

SR01_06_T2 SR01_07_T2 SR01_08_T2 SR01_09_T2 SR01_10_T2 SR01_11_T2 SR01_12_T2 SR01_13_T2

/MAXCAT=100

/CATEGORICAL=SQ04_01_T2 SQ04_03_T2

/EM(TOLERANCE=0.001 CONVERGENCE=0.0001 ITERATIONS=25).

*Scale mean, descriptives and reliability for BPS at T2.

COMPUTE BPS_T2_mean=MEAN (BP01_01_T2, BP01_02_T2, BP01_03_T2, BP01_04_T2, BP01_05_T2, BP01_06_T2, BP01_07_T2, BP01_08_T2, BP01_09_T2).

EXECUTE.

DESCRIPTIVES VARIABLES=BPS_T2_mean

/STATISTICS=MEAN STDDEV MIN MAX KURTOSIS SKEWNESS.

RELIABILITY

/VARIABLES=BP01_01_T2, BP01_02_T2, BP01_03_T2, BP01_04_T2, BP01_05_T2, BP01_06_T2, BP01_07_T2, BP01_08_T2, BP01_09_T2

/SCALE('ALL VARIABLES') ALL

/MODEL=ALPHA

/STATISTICS=DESCRIPTIVE CORRELATIONS

/SUMMARY=TOTAL.

*Bedtime at T2.

*Date and Time Wizard: Convert string into time variable.

COMPUTE SQ04_01_T2_time=number(SQ04_01_T2, TIME5).

VARIABLE LABELS SQ04_01_T2_time 'Anzahl geschlafener Stunden: Wann sind Sie w�hrend der letzten '+

'vier Wochen gew�hnlich abends zu Bett gegangen? ... �bliche Uhrzeit'.

VARIABLE LEVEL SQ04_01_T2_time (SCALE).

FORMATS SQ04_01_T2_time (TIME5).

VARIABLE WIDTH SQ04_01_T2_time(5).

EXECUTE.

*Correction for circularity: Bedtime after midnight + 24h.

IF (SQ04_01_T2_time < TIME.HMS(6,0,0)) SQ04_01_T2_time = SQ04_01_T2_time + TIME.HMS(24,0,0).

EXECUTE.

DESCRIPTIVES VARIABLES=SQ04_01_T2_time

/STATISTICS=MEAN STDDEV MIN MAX.

*Wake-up time at T2.

*Date and Time Wizard: Convert string into time variable.

COMPUTE SQ04_03_T2_time=number(SQ04_03_T2, TIME5).

VARIABLE LABELS SQ04_03_T2_time 'Anzahl geschlafener Stunden: Wann sind Sie w�hrend der letzten '+

'vier Wochen gew�hnlich morgens aufgestanden? ... �bliche Uhrzeit'.

VARIABLE LEVEL SQ04_03_T2_time (SCALE).

FORMATS SQ04_03_T2_time (TIME5).

VARIABLE WIDTH SQ04_03_T2_time(5).

EXECUTE.

DESCRIPTIVES VARIABLES=SQ04_03_T2_time

/STATISTICS=MEAN STDDEV MIN MAX.

*T2: Reported sleep duration T2 = real sleep duration T2?.

COMPUTE bedtime_sec_T2 = XDATE.TIME(SQ04_01_T2_time).

COMPUTE wake_up_sec_T2 = XDATE.TIME(SQ04_03_T2_time).

IF bedtime_sec_T2 > wake_up_sec_T2 bedtime_sec_T2 = bedtime_sec_T2 - 86400.

EXECUTE.

COMPUTE sleep_sec_T2 = (wake_up_sec_T2 - bedtime_sec_T2) - (SQ04_02_T2 * 60).

EXECUTE.

COMPUTE sleep_duration_calc_T2 = sleep_sec_T2 / 3600.

EXECUTE.

IF sleep_duration_calc_T2 > 14 sleep_duration_calc_T2 = $SYSMIS.

EXECUTE.

DESCRIPTIVES VARIABLES=sleep_duration_calc_T2

/STATISTICS=MEAN STDDEV MIN MAX.

CORRELATIONS

/VARIABLES=SQ04_04_T2 sleep_duration_calc_T2

/PRINT=TWOTAIL NOSIG FULL

/MISSING=PAIRWISE.

*Sleep latency at T2.

DESCRIPTIVES VARIABLES=SQ04_02_T2

/STATISTICS=MEAN STDDEV MIN MAX.

*Predictive validity.

*Bedtime at T2.

REGRESSION

/MISSING LISTWISE

/STATISTICS COEFF OUTS R ANOVA

/CRITERIA=PIN(.05) POUT(.10) TOLERANCE(.0001)

/NOORIGIN

/DEPENDENT SQ04_01_T2_time

/METHOD=ENTER BPS_mean.

*Wake-up time at T2.

REGRESSION

/MISSING LISTWISE

/STATISTICS COEFF OUTS R ANOVA

/CRITERIA=PIN(.05) POUT(.10) TOLERANCE(.0001)

/NOORIGIN

/DEPENDENT SQ04_03_T2_time

/METHOD=ENTER BPS_mean.
